# Forecasting the consumptions of coagulation tests using a deep learning model

**DOI:** 10.5937/jomb0-40244

**Published:** 2024-06-15

**Authors:** Basok Banu Isbilen, Kocakoc Ipek Deveci, Veli Iyilikci, Selena Kantarmaci, Mesut Fidan

**Affiliations:** 1 University of Health Sciences, Department of Medical Biochemistry, Tepecik Training and Research Hospital, Izmir, Turkey; 2 University of Health Sciences, Izmir Faculty of Medicine, Dr. Behçet Uz Child Disease and Pediatric Surgery Training and Research Hospital, Department of Medical Biochemistry, Izmir, Turkey; 3 Dokuz Eylul University, Faculty of Economics and Administrative Sciences, Department of Econometrics, Izmir, Turkey

**Keywords:** coagulation test, test consumption, test procurement, deep learning, artificial neural network, NARX (nonlinear autoregressive with external input) neural network, test koagulacije, potrošnja testa, nabavka testa, duboko učenje, veštačka neuronska mreža, NARKS (nelinearna autoregresivna sa eksternim ulazom) neuronska mreža

## Abstract

**Background:**

Laboratory professionals aim to provide a reliable laboratory service using public resources efficiently while planning a test's procurement. This intuitive approach is ineffective, as seen in the COVID-19 pandemic, where the dramatic changes in admissions (e.g. decreased patient admissions) and the purpose of testing (e.g. D-dimer) were experienced. A model based on objective data was developed that predicts the future test consumption of coagulation tests whose consumptions were highly variable during the pandemic.

**Methods:**

Between December 2018 and July 2021, monthly consumptions of coagulation tests (PTT, aPTT, D-dimer, fibrinogen), total-, inpatient-, outpatient-, emergency-, non-emergency -admission numbers were collected. The relationship between input and output is modeled with an external input nonlinear autoregressive artificial neural network (NARX) using the MATLAB program. Monthly test consumption between January and July 2021 was used to test the power of the forecasting model.

## Abstract (sr)

**Uvod:**

Laboratorijski stručnjaci imaju za cilj da obezbede pouzdanu laboratorijsku uslugu koristeći javne resurse efikasno dok planiraju nabavku testa. Ovaj intuitivni pristup je neefikasan, kao što se vidi u pandemiji COVID-19, gde su doživljene dramatične promene u prijemu (npr. smanjen broj prijema pacijenata) i svrha testiranja (npr. D-dimer). Razvijen je model zasnovan na objektivnim podacima koji predviđa buduću potrošnju testova koagulacionih testova čija je potrošnja bila veoma varijabilna tokom pandemije.

**Metode:**

Od decembra 2018. do jula 2021. prikupljane su mesečne potrošnje testova koagulacije (PTT, aPTT, D-dimer, fibrinogen), ukupnih, bolničkih, ambulantnih, hitnih, nehitnih prijemnih brojeva. Odnos između ulaza i izlaza je modelovan sa eksternom ulaznom nelinearnom autoregresivnom veštačkom neuronskom mrežom (NARKS) korišćenjem MATLAB programa. Mesečna potrošnja testova između januara i jula 2021. korišćena je za testiranje snage modela predviđanja.

**Rezultati:**

Prema kointegracionoj analizi, u model je uključen ukupan broj kao i broj hitnih i nehitnih pregleda i broj radnih dana u mesecu. Kada je procenjena potrošnja aPTT i fibrinogena, bilo je moguće predvideti potrošnju drugih testova. Podaci za pedeset meseci korišćeni su za predviđanje potrošnje u narednih šest meseci, a predviđanje zasnovano na NARKS-u bilo je robusniji pristup za oba testa.

**Zaključak:**

Model dubokog učenja daje bolje rezultate od intuitivnog pristupa u predviđanju, čak i u eri pandemije, i pokazuje da će efektivnije i efikasnije planiranje biti moguće ako se u predviđanju koriste mehanizmi odlučivanja podržani ANN-om.

## Introduction

Laboratory experts aim to provide reliable and high-quality laboratory service by efficiently using public resources (planning test procurement) [1]. Methods to be followed in test procurement processes differ in the public and private sectors [2]. While public tender or procurement processes and test/device purchases carried out in the public sector are in line with the legislation of the Public Procurement Authority, it is compared with the practices in the form of a direct agreement between the buyer-contractor in private sector procurement.

Regardless of procurement processes, one of the first steps in the test/device procurement process in the public and private sectors is to plan well and forecast the quantity of each test/device to be purchased for a certain period in the future [3]. This prediction is necessary to meet needs in a timely manner, and fewer or more tests should not be purchased. In the International Organization for Stan dardization 15189 Medical Laboratories-Require ments for Quality and Competence standard, it is stated that laboratories should have a documented procedure for equipment selection, purchase, and equipment management [4]. In practice, the total number of tests, as well as the test number of each test are required to present in the public tender document, and no procedure for the forecasting of test consumption is specified in terms of the Turkish Public Procurement General Communiqué [5]. As it can be seen, there is no legislation or regulation for test consumption estimation, which can change according to the test/device whose consumption is planned, the institution it is taken, the purpose of use, and it proceeds more intuitively according to the experience of laboratory specialists, both on an international and national basis. The conventional method used in practice is the first average of the past monthly consumption is determined, next the annual consumption amount is calculated, and the last annual need for a test is estimated by adding at least a 10% increase for each year of the purchase.

Evidence-based medicine is required objective data, and one of the fundamental sources providing the data in medicine is laboratory medicine. The role of laboratory medicine in the COVID-19 pandemic extends beyond initial diagnosis and epidemiological surveillance [6]. Due to the essential role of laboratory service in assessing the COVID-19 disease severity, selecting appropriate therapeutic options, and monitoring treatment response, routine biochemical, hematological, and immunochemical test orders fluctuated during the pandemic era. Specifically, the number of coagulation test panels consumed, including activated partial thromboplastin time (aPTT), prothrombin time (PT), fibrinogen, and D-dimer, fluctuated over the period. In our experience, D-dimer orders increased six times, fibrinogen three times, aPTT, and PT tests increased two times. Undoubtedly, it is not probable to detect these temporal changes in the tender planning made before the pandemic. This process, in which test request management is very challenging due to the inadequacy of current projections, has revealed the incompetence of the conventional method in test consumption forecasting. Using more rational planning approaches that include such periods, which are extremely challenging for laboratory and institution managers, it might be possible to be more prepared for potential new crisis periods.

Machine learning and artificial intelligence (AI) applications help reduce costly, time-consuming manual processes, and change the cost-quality curve in healthcare [7]. AI is utilized heavily for a wide range of healthcare management applications, including clinical reporting, patient communication and management, payment administration, management of sales cycles, and management of medical records. AI-based technologies, including deep learning (DL), are being employed in predictive analytics to aggregate and analyse disparate data types, recognize patterns, and trends within that data, and make more informed decisions to pre-emptively alter future outcomes [7].

Lean laboratory management focuses on clearing all the steps and activities that do not add value that compels the laboratory workflow, that is, waste [8]. Since the intuitive approach is based only on past test consumption, it forces the provision of lean laboratory management, especially in times of crisis, because it hinders workflows. The conventional method is inadequate as seen in the COVID-19 pandemic, where the dramatic changes in admissions (decreased patient admissions) and the purpose of testing, such as observed in D-dimer orders have been experienced. In this study, we aimed to more efficiently determine future test consumption in crisis periods when temporal fluctuations in test consumption are large, such as in a pandemic, with the DL model (NAR ) as an alternative to the intuitive approach.

## Materials and methods

Monthly based consumptions of PT, aPTT, D-dimer, and fibrinogen tests, and number of total-, inpatient-, outpatient-, emergency-, and non-emergency examinations per month were retrospectively collected between December 2018 and July 2021. PT, aPTT, fibrinogen, and D-dimer levels were analyzed in the Sysmex CS2500 (Sysmex Inc., Kobe, Japan) coagulation device. The variables to be included in the model were determined by cointegration analysis. The relationship between inputs and outputs was modeled with the nonlinear autoregressive artificial neural network (ANN) with external input (NARX) by using MATLAB version R2022b (MathWorks, Natick, MA, USA). Monthly test consumptions between January-July 2021 were used to test the models’ prediction power.

In this study, NARX models are utilized for modeling the non-linear dynamical nature of the data. NARX is a variant of recurrent neural networks and a preferred method, especially for time series modeling. In NARX prediction, the future values of a time series are predicted from past values of that series and other external time series. NARX networks are used in prediction studies where the desired output depends on the data in the past [9]. Recurrent neural networks are a class of neural networks in which previous outputs can be used as inputs. Unlike other recurrent neural networks, NARX has feedback links that span several layers of the network. To make successful predictions in non-linear time series, the memory capability of real or predicted previous values is used in this method. A NARX network can gain degrees of freedom when it includes a time period forecast as an input for subsequent periods compared to a feedforward network [10].

NARX consists of two layers of feedforward network, input, hidden layer and output layer, and output. It also includes biases (b), input weight (IW), and layer weight (LW) values. F1 is the activation function in the hidden layer, and f_2_ is the activation function of the output layer. For f_1_ in the hidden layer, the sigmoid function is used to scale the inputs in the network in the range (-1, 1) and reduce the error rate when applying backpropagation calculations. The linear function is used for f_2_ in the output layer. Initially, the weights are randomly determined. In the training process, these weights are rearranged so that the output value produced by the ANN is close to the target value.

Before starting the training, it is necessary to determine the input and feedback time delays, as well as the size of the hidden layer, the type of feedback, and the training function. NARX includes two types of feedback, parallel (closed-loop) and series-parallel (open loop). In the parallel type of NARX design, the predicted target value feeds back into the time delay line of the feedforward neural network.

Various performance measures can monitor NARX training performance and minimize errors. Adjustments are made on weights and biases to reduce mean square error as quickly as possible. One of the methods used for this process is the Bayesian regulation (BR) method in which, there is a probability distribution of the weights, unlike the traditional training method, where the weights are selected in a way that minimizes the error function. In this case, the estimation of the network is the result of a probability distribution. Training with BR takes longer but is more successful for small datasets. In the study, the BR algorithm was adopted as training algorithm while using the NARX model.

First the data is formed as input and output matrices. 80% of the data set was used for training, 10% for validation, and 10% for testing. The NARX model used in the study has 2 delay steps, 10 neurons in the hidden layer, and 1 neuron in the output layer. After training the model for each of the outputs (PT and aPTT), remaining data is used for validation and testing. The performance measures of the models are also calculated. The codes and data are available in the following GitHub repository: https:// github.com/ipekdk/narx-code.

## Results

**Figure 1:**
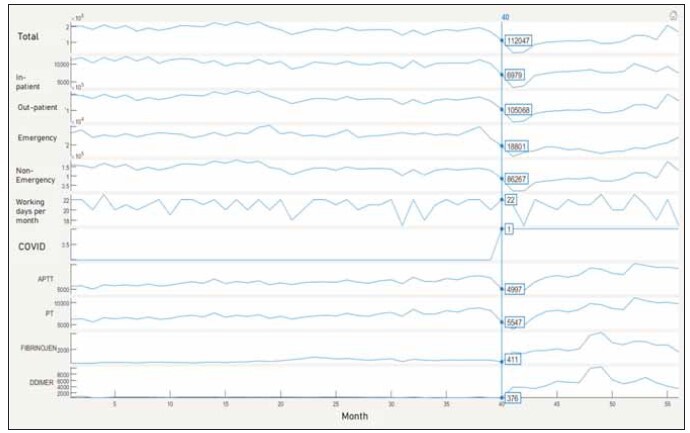
Data per month for each parameter. Top to bottom on y-axis: Total-patient-, inpatient-, outpatient-, emergency-, non-emergency- admission counts, working days per month, COVID absence or presence, activated partial thromboplastin time (aPTT), prothrombin time (PT), fibrinogen, and D-dimer consumptions.

According to the results of the cointegration analysis, the number of total-, emergency-, and non-emergency examinations plus the number of working days per month are recommended to be included in the model. As can be seen from Figure 1, the consumption trends of PT and aPTT tests were parallel, and a similar trend was also present between consumption of fibrinogen and D-dimer. Once aPTT and fibrinogen consumption have been estimated, then consumption of PT and D-dimer tests can be similarly estimated. So, two NARX models were trained for these variables. One model has aPTT usage and the other one has fibrinogen usage as the dependent variable. Fifty months of test consumption data were used to predict test needs over the next six months.

R-value represents the fit of the model estimation to real values. While the training, test, and all observation R-values were 0.988, 0.954, and 0.967 for the aPTT model, the R-values were 0.999, 0.994, and 0.987 for the fibrinogen model, respectively. The R values of training, test and all observations for both tests were very high (close to 1) indicating a good fit to the data. The NARX model for the fibrinogen assay had a better fit.

**Figure 2:**
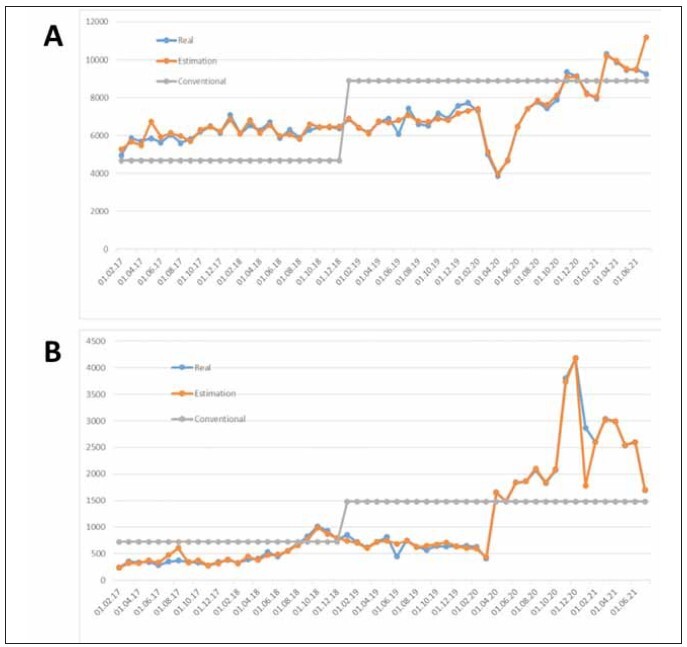
(A) Comparison of real-time, estimated (deep learning approach (DL)), and conventional (intuitive approach) test usage for aPTT, (B) Comparison of real-time, estimated (DL approach), and conventional (intuitive approach) test usage for fibrinogen.

Data for the first 50 months were used for model training and validation, and data for the last 6 months were used for model evaluation. Actual and estimated values for both tests are given in Figure 2A and Figure 2B, consecutively. It can be seen that both models have very satisfactory estimations. Estimated values are very close to real values for most of the months. When only past values of the test usage were utilized as a time series model, the estimation would not be that good. Since the NARX model permits the inclusion of external variables (total-, emergency-, non-emergency-examination numbers, and the number of working days per month) and their lagged values for use in model training, the model’s estimation power is significantly increased.

As seen in both Figure 2A and Figure 2B, the intuitive approach performs insufficiently by over or underestimating tests from time to time compared to actual consumptions and DL-supported model estimations.

## Discussion

In this study, a DL model that can incorporate external variables into a time series analysis is used for predicting the numbers of two coagulation tests, aPTT, and fibrinogen. The predictive power of the models was assessed by developing a model that predicts future consumption of coagulation panel tests. Consumption of these tests was highly variable during the pandemic period, based on objective data. The DL model gives better results than the intuitive approach in terms of forecasting, even in the pandemic era, and it shows that more effective and efficient planning will be possible if ANN-supported decision mechanisms are used in forecasting tests’ consumptions in the procurement process.

Some studies predict future spending in health services (such as the number of patient visits in the emergency department or the number of critical services such as intensive care) by evaluating drug distribution in pharmacies to determine effective inventory levels or by estimating non-adherence to appointments. As in our example, there are no models for determining test consumption. In general, healthcare administrators often make short-term forecasts to manage day-to-day operations within their organizations to minimize variability. However, short-term planning of laboratory test procurement processes makes it difficult to sustain the laboratory workflow, and therefore procurement processes are tried to be planned by making medium-term forecasts. However, these forecasts are often based on human intuition and a minimally scientific assessment of contributing factors.

Using machine learning estimations in health service planning, such as test procurement processes or patient examination can enable more rational and efficient planning. In a study using the convolutional neural networks method for the estimation of the number of patients examinations to the emergency department, it was shown that the DL model (PatientFlowNet) achieved better accuracy than the current state-of-the-art models in estimating patient flow. In a study from our country, Esen and Kaya estimated the number of patient examinations to the Emergency Service using the random forest, which is a machine learning model, and Holt-Winters models [9]. In the first model, the model was developed over the variables of the number of patients, the population of the city, and the number of tourists, while in the Holt-Winters model, the model was built on the number of past applications. It has been determined that the Holt-Winters Method fits seasonal data better and is more convenient than the random forest model. Regardless of the model used and the variable to be estimated, it has been shown that predictions made from past data in health services are more successful than state-of-art approaches. In our model, laboratory test consumptions were forecasted instead of patient examination and provided more accurate results than the conventional approach.

Monthly data before December 2018 were not included in the study as it was intended to have the data which was produced by the same instrument and methodology. This is a limitation because, as with the intuitive procurement approach, the DL-model needs to estimate test consumption regardless of device or method. Since the primary purpose of this study is to develop a new model for the test consumptions, how the device and method change will affect the DL-model has not been evaluated.

Monthly data before December 2018 did not include in the study to have the test results analysed by the same instrument and methodology. It might be considered a limitation because, the DL-model needs to estimate test consumption regardless of device or method. Since the primary purpose of this study is to develop a novel model for the test consumptions, how the device and method change will affect the DL-model has not been evaluated.

In order to predict service needs and effectively use resources over time, it is critical for decision makers to have accurate data assessments in health care systems. For the sustainability of health care, the consistency of the assessments made, especially during periods of health crises such as pandemics, is extremely necessary. Using such predictive applications (AI-based) gives decision makers the ability to predict service needs and make the right decisions when managing resources.

## Dodatak

### Acknowledgement

Selena Kartarmaci has a grant from the Council of Higher Education scholarship program that is 1000/2000 Artificial Intelligence and Machine Learning.

### Conflict of interest statement

All the authors declare that they have no conflict of interest in this work.
